# Supplementary material for: Assessment of the role of anthocyanin standardized elderberry (Sambucus nigra) extract as an immune-stimulating nutraceutical of Nile tilapia, Oreochromis niloticus
Source: PLoS One. 2022 Dec 30;17(12):e0279471. doi: 10.1371/journal.pone.0279471 (PMC9803303; doi:10.1371/journal.pone.0279471)
Supplement: Attachment — Submitted filename: Response To Reviewers.docx [file pone.0279471.s002.docx]

Ref: PONE-D-22-27453

RESPONSE TO REVIEWERS

We would like to thank the editor and the reviewers for their kind considerations and suggestions to improve the manuscript.

Following the suggestion of the reviewers/editor:

The manuscript has been edited to meet the criteria and formatting of PLOS ONE. The methods section was edited, and the manuscript now contains a separate ethics statement including the name of the ethics body and the approved protocol number. The references were edited to meet the journal formatting requirements. General grammatical errors and spellings have been corrected.

Response to reviewers

To Reviewer #1: Thank you for reading our work! We appreciate your feedback.

To Reviewer #2: Thank you for your feedback! Following your suggestions, we have now edited the introduction to include the information that the Elderberry under study is of the Austrian variety. The methods section was edited to include further details of the extraction process by the manufacturer to clarify the description of the elderberry extract as stated in the literature accompanying the product.

We believe these changes will make the manuscript better and we are thankful for the suggestions. Please let us know if there are any other suggestions, improvements to be made or we missed.
